# Interactive Computer-Adaptive Chronic Kidney Disease (I-C-CKD) Education for Hospitalized African American Patients: Protocol for a Randomized Controlled Trial

**DOI:** 10.2196/66846

**Published:** 2025-04-17

**Authors:** Akilah King, Tayo Omoniyi, Lindsay Zasadzinski, Cynthia Gaspard, Denesha Gorman, Milda Saunders

**Affiliations:** 1 Section of General Internal Medicine Department of Medicine University of Chicago Chicago, IL United States

**Keywords:** chronic kidney disease, computerized adaptive education, end-stage kidney disease, end-stage renal disease, glomerular filtration rate, kidney failure, usual hospital care, inpatient

## Abstract

**Background:**

End-stage kidney disease (ESKD) or kidney failure is a condition where the kidneys lose the ability to function. African American individuals are 4 times as likely to develop ESKD compared to White American individuals. In addition, African American patients are less likely to have an optimal dialysis start and to choose renal replacement therapy modalities that align with their goals and values. Our prior work shows that culturally tailored, in-person education improves patient outcomes. This is the foundation for our innovative intervention using an African American virtual patient educator as an option for hospitalized patients with chronic kidney disease (CKD).

**Objective:**

The Interactive Computer-Adaptive Chronic Kidney Disease (I-C-CKD) study will determine whether the computerized adaptive education and usual hospital care impact the health literacy of African American patients with kidney disease. It will also assess how patients’ lifestyle and commitment to health goals are impacted by the method of health literacy education.

**Methods:**

We will screen, recruit, and enroll hospitalized patients who self-identify as African American and have advanced CKD based on their estimated glomerular filtration rate. Eligible patients who verbally consented will be randomly assigned into either the computerized adaptive education intervention group or the control group (usual hospital care). Patients in the intervention group will receive a culturally tailored, adaptive education module. To analyze pretest, posttest, and follow-up survey results on patient CKD knowledge, ESKD treatment options, and health goals, we will use a paired, 2-tailed *t* test with a Bonferroni adjustment for multiple comparisons.

**Results:**

Recruitment for the I-C-CKD study began on May 2, 2023. We are currently recruiting and have enrolled 96 patients who completed both pretest and posttest surveys as of December 2024. This includes 50 patients in the control group and 46 patients in the intervention group. Data analysis has not occurred.

**Conclusions:**

African American individuals often receive less patient education about self-care and treatment options for CKD. We hope this study provides a solution to increase hospitalized African American patients’ knowledge of CKD and motivation for CKD self-care through computerized adaptive education, reduce disparities, and improve patient outcomes.

**Trial Registration:**

ClinicalTrials.gov NCT06364358; https://clinicaltrials.gov/study/NCT06364358

**International Registered Report Identifier (IRRID):**

DERR1-10.2196/66846

## Introduction

### Background

End-stage kidney disease (ESKD) or kidney failure is a condition where the kidneys lose the ability to function. African American individuals are 4 times as likely to have ESKD compared to White American individuals [1]. African American patients are more likely to have comorbidities such as diabetes, obesity, high blood pressure (hypertension), and heart failure, which all increase the risk of developing kidney disease [1].

Hemodialysis can help patients with ESKD feel better and live longer. However, dialysis is not a cure for ESKD, and patients on hemodialysis have high morbidity and mortality rates [1]. Patients on hemodialysis can also experience side effects such as low blood pressure (hypotension), muscle cramps, sleep problems, anemia, bone disease, high blood pressure (hypertension), and access-site complications [2]. In-center hemodialysis (IHD) with thrice weekly sessions in a dialysis unit is the most common treatment for ESKD. Patients who use IHD experience greater inconvenience, worse waste clearance, and more issues with transportation in comparison to other ESKD options. Alternatives to IHD include home hemodialysis, peritoneal dialysis, and kidney transplantation. These methods are associated reduced morbidity and mortality, or improved quality of life, compared to IHD [3].

There are racial disparities in the use of these renal replacement therapy (RRT) modalities. African American patients are less likely to be informed about better RRT options. African American patients are 47% less likely to use peritoneal dialysis and 3.5 times less likely to have a kidney transplant [4]. Often, African American patients are less likely to receive information from their provider about their CKD stage, treatment options, and self-care to prevent and treat CKD [3]. The hospital can be an important intervention site because hospitalization rate rises sharply in the 3 months before dialysis initiation. In addition, even patients who are not connected to primary or specialty care can present to the hospital.

Our previous work to was to develop and test a hospital-based CKD intervention, PREP RRT (Patient Referral and Education Program Prior to Renal Replacement Therapy). To develop the intervention, we interviewed African American patients about CKD knowledge and barriers to RRT preparation [4,5]. The intervention in that study includes an in-person African American health educator and written materials [2]. Our results showed an increase in knowledge in the intervention group compared to control group. We also found that culturally tailored interventions improved patient outcomes [6]. Drawing from insights gained in our prior work, our research team developed and implemented the Interactive Computer-Adaptive Chronic Kidney Disease (I-C-CKD) education as a patient education intervention to inform hospitalized African American patients with advanced CKD about their CKD and RRT options. This computer-based education is predicted be more accessible for hospitalized patients through videos that are culturally tailored with a virtual African American narrator, as in-person educators have some limitations.

### Primary Hypothesis

Our primary hypothesis is that the computerized adaptive education will be more effective than usual hospital care in improving knowledge about CKD, CKD self-care, and RRT options (primary outcome).

### Secondary Hypothesis

Our secondary hypothesis is that the computerized adaptive education will increase patients’ intent to participate in CKD self-care compared to usual hospital care. We also believe that the computerized adaptive education will increase patients’ action and commitment to CKD health-seeking behavior, access planning prior to dialysis initiation, initiate home dialysis modalities, and/or have a transplant evaluation after discharge and beyond 30-day follow-up.

## Methods

### Trial Design

The I-C-CKD study is a hospital-based, randomized controlled trial of a computerized patient education intervention compared to usual hospital care. After obtaining verbal consent (Multimedia Appendix 1), each patient will be randomly assigned into two groups: usual hospital care (control group) and the computerized adaptive education (intervention group), which is assisted by a research assistant (RA).

The usual hospital care control group will feature a baseline General Health Knowledge and Intent survey. Participants in the usual hospital care control group will receive the pretest survey and education materials but will not receive specific knowledge about CKD or their CKD condition. Participants in the control group will receive printed patient education materials informing them of the importance of a general healthy lifestyle, including diet, physical activity, and medication adherence. The printed materials for the usual hospital care control group are meant for patients with cardiovascular conditions and do not have any information about CKD. Participants who receive the control condition will also be able to speak with the patient educator if they want to.

**Figure 1:**
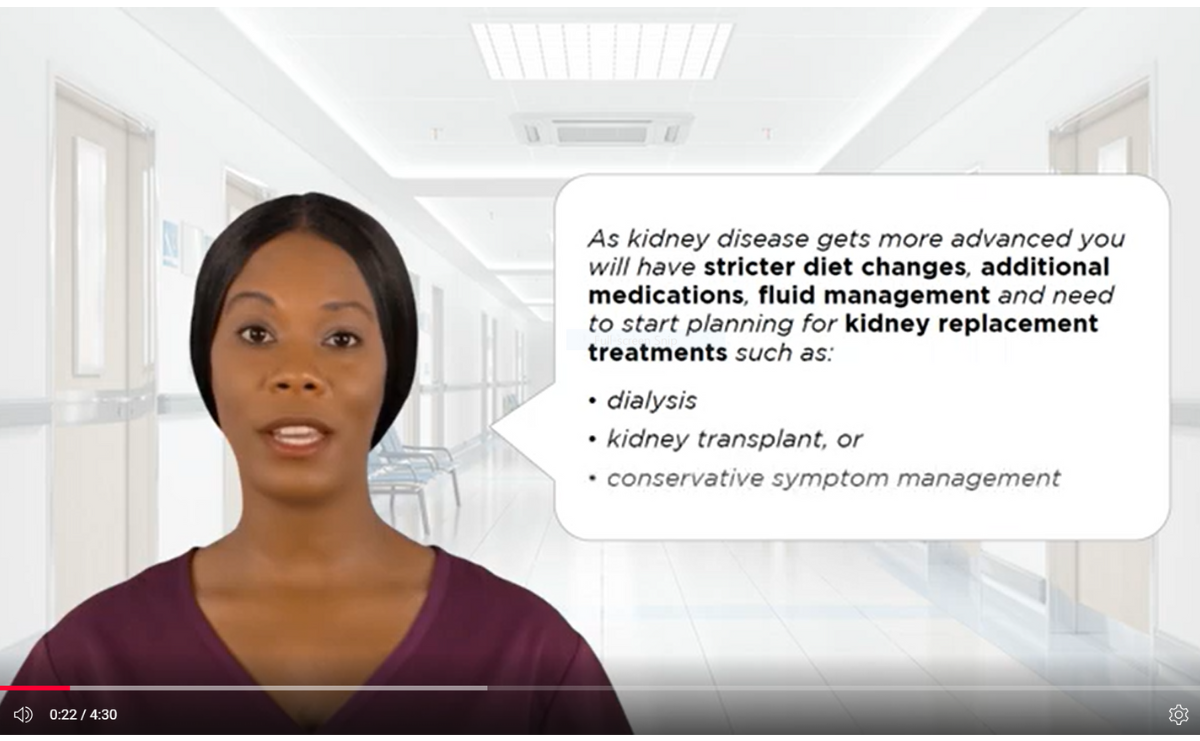
Screenshot of the computerized adaptive education intervention featuring a personal story.

**Figure 2:**
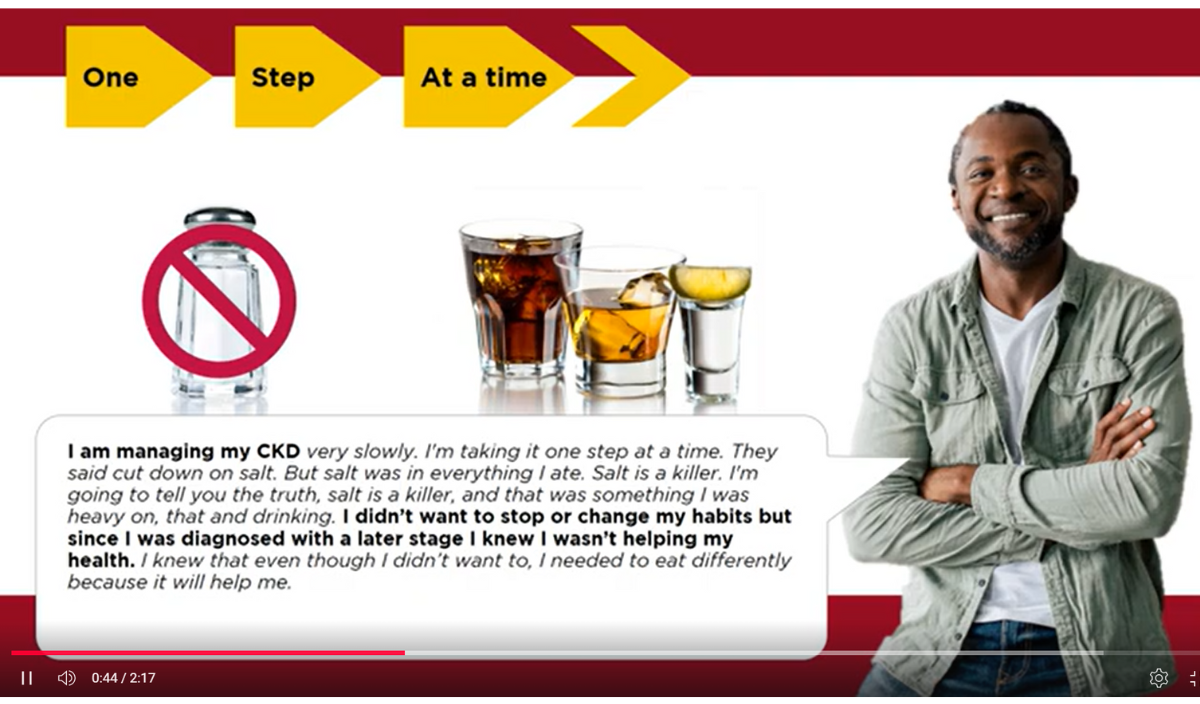
Screenshot of the computerized adaptive education intervention regarding treatment options. CKD: chronic kidney disease.

The computerized adaptive education intervention group will receive the advanced CKD education module with information on the risk factors, stages, treatment options, and lifestyle of patients with CKD (see Figures 1 and 2). The RAs will assist with the program but not offer any information or knowledge about CKD. Following the patient’s review of the advanced CKD education module, the RAs will administer the posttest General Health Knowledge and Intent survey.

Both the usual hospital care control group and the computerized adaptive education intervention group will complete the “What’s Next” form, to assess the patient’s commitment to specific self-care activities. Patients will select their top-3 health goals, rate how confident they are about each goal, and identify how important each goal is to them. Patients will also report on their health resources. All the forms will be sent to the patient educator, a trained nephrology social worker.

Each patient will be contacted 30 days after discharge to ask about their commitment to health goals, barriers to self-care, and treatment plans.

The SPIRIT (Standard Protocol Items: Recommendations for Interventional Trials) checklist is provided in Multimedia Appendix 2.

### Participants and Eligibility

The study will take place at the University of Chicago Medical Center (UCMC). We will approach hospitalized, English-speaking, African American patients at UCMC, aged 18-70 years, who meet the criteria for advanced CKD (Stage 3b or greater) with an estimated glomerular filtration rate (eGFR) <45 mL/min/1.73 m^2^; they must not be on dialysis yet or have a history of kidney transplant. In order to be eligible, patients must have an eGFR <45 mL/min/1.73 m^2^ during the current hospitalization in addition to prior hospitalizations and/or outpatient visits. We will have access to prior UCMC inpatient and outpatient laboratory values. We will also have access to some inpatient and outpatient laboratory values from other medical centers through Epic Care Everywhere. The exclusion criteria include patients who are hospitalized in the intensive care unit, meet the Montreal Cognitive Assessment criterion for cognitive impairment, or require a proxy for study consent. We will enroll 170 participants and randomly assign 85 to the computerized adaptive education and 85 to usual hospital care.

### Recruitment

This study will be a substudy of the Hospitalist Project, an ongoing clinical study that examines a variety of outcomes of patients hospitalized in our general internal medicine services, and includes the analysis of administrative data, inpatient interviews, and 30-day follow-up by phone [7]. Hospitalist Project staff will obtain basic demographic information and medical history. Per the Hospitalist Project protocol, staff will also obtain permission to contact patients after discharge and to access their medical records (ie, at UCMC, from other medical systems, and through Medicare linkage). All inpatients recruited to the Hospitalist Project will be screened daily by the RA to identify patient eligibility for the study.

We will approach all Hospitalist Project–enrolled patients who meet study criteria and obtain informed verbal consent.

### Randomization

Participants will be randomly assigned using a computerized spreadsheet, assigning each participant a random number. Participants who are assigned an odd number will be assigned to the intervention group. Participants who are assigned an even number will be assigned to the control group. The RA will begin by identifying an eligible participant who consented to the Hospitalist Project. Then, they will use the randomization spreadsheet to determine if the participant will be part of the intervention group or the control group. They will then approach the patient with the appropriate form and study packet.

The RA will complete the randomization, assignment, and enrollment process.

### Blinding

Trial participants will be blinded after group assignment. Participants will view the survey as a Google Form and will not be informed whether they are part of the intervention or control group. They will also receive a study packet, which will not identify if they are part of the intervention or control group. The research team will not be blinded.

### Intervention Group

The RA will meet each participant and conduct the pretest survey in the participant’s private hospital room. The computerized adaptive education intervention group will receive an advanced CKD education module with information about CKD risk factors, stages, treatment options, and lifestyle modifications. The RA will assist in advancing program modules but not offer any information or knowledge about CKD. The avatar will be an African American woman who will present statistics about African American individuals and CKD. The interactive component will allow participants to personalize the information received based on prior CKD knowledge, diabetes status, and the level of motivation. The interactive CKD video can be completed in 30-45 minutes. Following the patient’s completion of the advanced CKD education module, the RA will then readminister the General Health Knowledge and Intent survey.

The computerized adaptive education intervention group (and the usual health care control group) will complete the “What’s Next” form, to assess the patient’s commitment to specific self-care activities. Patients will select 3 health goals from a list of 10 options, rate their level of confidence in the ability to make change, and identify an action plan to accomplish each goal. Moreover, patients will be asked to report their needs from the medical care team and for community assistance, and they will be provided a resource list. The study will also ask if patients would like a primary care doctor and/or a nephrologist. This information will be relayed to the primary inpatient team. All the forms will be sent to the patient educator, a trained nephrology social worker.

We will encourage participants to complete the voluntary study in one visit. At 30 days after discharge, both groups will complete a follow-up call with the project RA. Participants will be queried about progress on goals set on the health commitment form, knowledge, and health intent.

### Control Group

The RA will meet each patient and conduct the pretest survey in the participant’s private hospital room. The usual hospital care control group will receive the General Health Knowledge and Intent pretest survey and printed education materials without explicit CKD information or knowledge about their CKD condition. The distributed education materials were intended for patients with cardiovascular conditions and focus on the importance of a general healthy lifestyle, including diet, physical activity, and medication adherence, and does not include CKD specific information. Following the patient’s completion of the general health education video module, the RA will then readminister the General Health Knowledge and Intent survey.

The usual hospital care control group (and the computerized adaptive education intervention group) will complete the “What’s Next” form, to assess the patient’s commitment to 3 participant-selected self-care activities. Participants also will be contacted 30 days after discharge to complete the follow-up survey.

### Outcomes

#### Primary Outcome

The primary outcome of this study is the changes in patient knowledge, attitudes, and behavior about CKD and RRT. To evaluate CKD and CKD self-care knowledge, we will use a modified Kidney Knowledge Survey; the CKD Self-Management Knowledge Toolkit, which is a validated instrument [8,9]; and an investigator-developed ESKD knowledge survey. We will measure the change from the baseline observed via the pretest, posttest, and follow-up surveys. The pretest and posttest surveys comprise 20 mixed format multiple-choice and Likert-scale questions (ranging from strongly agree to strongly disagree; from no knowledge to a great deal of knowledge; and from extremely unlikely to extremely likely) to examine the patient’s basic understanding of their health, general CKD knowledge, and health management.

#### Secondary Outcomes

The secondary outcomes are patient motivation and intent. We will administer the CKD Self-Efficacy Scale [10], the Patient Activation Measure [11], and the Kidney Failure Treatment Preferences [12] before the intervention, immediately after the intervention, and at 30 days after discharge. We will administer the General Health Knowledge and Intent survey, an investigator-developed instrument, to assess RRT preferences and commitment to CKD self-care activities, including intent to seek a nephrologist, noncatheter dialysis access initiation, and completion of a transplant evaluation. Each patient will be contacted 30 days after discharge to discuss their commitment to each selected health goals, barriers, and treatment plan.

### Data Collection and Management

We will retrieve baseline survey information from the Hospitalist Project, which includes patient demographics and comorbidities. The initial trial data will be collected, stored, and saved in Google Forms with linked Google Sheets for the pretest and posttest surveys on the computer. The RA will photograph and save participants’ written packet responses digitally. The RA will deidentify the packet responses and save them according to their initials, study visit date, and enrollment number. We will ensure participant responses remain confidential. Information about the patient’s medical status, enrollment, and participation will be maintained through a password-encrypted file.

The RA will use password-encrypted laptops to conduct the study. The participant’s responses on the study packet will be photographed, deidentified, and saved in a password-encrypted digital folder. The analysis file will not be attached to protected health information to protect patient privacy. We will conduct analysis in a locked office with a password-protected computer. All transcripts and surveys will be deidentified.

### Statistical Methods

The planned total sample size is 170 (85 per group), which accounts for participant dropout due to unexpected discharges, changes in clinical status, or study disenrollment. The primary outcome will be measured by the change in baseline observed via the pretest, posttest, and follow-up surveys. Using a 2-sample, 2-tailed *t* test, a total of 154 (77 per group) participants is required to obtain >90% power to detect at least a 10% difference with the most conservative SD of 21% with a 1-sided type-1 error of 5%.

Our team will use the paired, 2-tailed *t* test with a Bonferroni adjustment for multiple comparisons with the usual hospital care and computerized adaptive education groups. We will use one-way ANOVA and chi-square tests to examine the significance between the usual hospital care and computerized adaptive education groups. This will determine whether there is a statistically significant difference between the two groups. We will also determine if the difference between observed and expected data is because of chance or a relationship between the usual health care and computerized adaptive education groups.

Data analysis will take place in an UCMC office. We will continue to monitor patient medical records after the study to determine whether they may be later included or excluded from the study.

### Data Tracking

Data will be tracked in Google Sheets, linked to each survey. This will also help identify participant’s responses with the date, time, and enrollment number. Study packet responses will be saved in a password-protected box. Retrospective review will also be done to ensure each enrollment number matches the correct medical record number. Our research team will have access to the final trial dataset, and we will keep the data for 5 years after the study is completed.

### Ethical Considerations

We obtained institutional review board (IRB) approval for this protocol (IRB 23-0385), and the parent study (the Hospitalist Project) is also IRB approved (IBR 16-1131). Written, informed consent to participate in the Hospitalist Project will be obtained from all participants. Participants will also verbally consent and be enrolled in the I-C-CKD study.

Patients will be informed that the study is completely voluntary and may withdraw at any time. Discontinuation will also occur if patients are in acute distress or experience declining health conditions during hospitalization. For example, if the patient is transferred to intensive care unit, is intubated, or has an altered mental status and can no longer participate, then we will withdraw them from the study. Patients will also be able to meet with patient educator who is a licensed clinical social worker if they are interested in additional information, are under distress, or want posttrial care.

Patients will receive US $25 gift cards upon completion of the pretest and posttest surveys and US $10 for partially completed surveys. All participants will be compensated. This study is minimal risk, as an education intervention is the only experimental procedure involved. Additionally, reports will be made to the IRB in the case of an adverse event, along with any protocol modifications to the investigators, IRB, trial participants, journals, and regulators.

## Results

Recruitment for the I-C-CKD study began May 2, 2023. We are currently recruiting and have enrolled 96 patients who completed both pretest and posttest surveys as of December 2024. This includes 50 patients in the control group and 46 patients in the intervention group. Data analysis has not occurred. We will communicate trial results to trial participants, health care professionals, and the public via a journal publication.

## Discussion

We believe our study will result in the computerized adaptive education intervention group experiencing a greater improvement in CKD knowledge, CKD health-seeking behavior, and self-care in comparison to the control group. The goal of this study is to increase hospitalized African American patients’ knowledge of CKD and motivation for CKD self-care. We have created and will assess a culturally tailored, computerized adaptive education intervention that expands on the information gathered from in-person interventions. Computerized adaptive education can evaluate patients’ concerns and provide personalized education. Customized patient education information is more likely to be recalled and significantly influence a patient’s motivation and behavior modification.

African American patients diagnosed with CKD experience inequities in the quality of health education, self-care, and medical treatment for ESKD [13]. Patient education can encourage African American patients with advanced-stage CKD to make informed decisions and take control of self-care and health decisions to delay progression to ESKD. Providing CKD education during hospitalization can address gaps in care to help patients initiate preventative care, plan for future RRT, and build a treatment team that aligns with their health and lifestyle preferences.

This intervention focuses on hospitalized African American patients who represent more than 80% of the general medicine population at our medical center. The intervention is culturally tailored to African American patients. The hospital also serves patients who are not followed by routine medical providers and have trouble accessing outpatient care due to limited transportation, limited or no insurance, mobility issues, and socioeconomic constraints [14]. Hospitalized patients may be more open and responsive to CKD education efforts when they are focused on their health during a hospital stay. The intervention can also link these patients to outpatient care. We will also ask if patients would like a primary care doctor and/or a nephrologist. This information will be relayed to the primary inpatient team.

Limitations of this study include the single-hospital study design, which reduces the generalizability of the results. In addition, we are testing the intervention only among hospitalized African American patients who are generally admitted with urgent or acute health concerns, which may reduce their ability to fully participate or complete this study. An additional barrier for inpatient kidney education to be disseminated on a large scale is that the results may differ among a different group or in a different health care setting.

In conclusion, African American individuals often receive less patient education about self-care and medical treatments for CKD. We hope our study provides one way to reduce this disparity. However, to improve CKD outcomes, African American patients—and all patients with CKD—require frequent, understandable education about CKD across all sites of care.

## Appendix

Multimedia Appendix 1 Institutional review board–approved consent form.

Multimedia Appendix 2 SPIRIT (Standard Protocol Items: Recommendations for Interventional Trials) checklist.

## Abbreviations

CKD: chronic kidney disease
eGFR: estimated glomerular filtration rate
ESKD: end-stage kidney disease
I-C-CKD: Interactive Computer-Adaptive Chronic Kidney Disease
IHD: in-center hemodialysis
IRB: institutional review board
PREP RRT: Patient Referral and Education Program Prior to Renal Replacement Therapy
RA: research assistant
RRT: renal replacement therapy
SPIRIT: Standard Protocol Items: Recommendations for Interventional Trials
UCMC: University of Chicago Medical Center

## Data Availability

The datasets generated or analyzed during this study are not publicly available as data analysis has not occurred at this time but are available from the corresponding author on reasonable request.
