# Supplementary material for: Interactive Computer-Adaptive Chronic Kidney Disease (I-C-CKD) Education for Hospitalized African American Patients: Protocol for a Randomized Controlled Trial
Source: JMIR Res Protoc. 2025 Apr 17;14:e66846. doi: 10.2196/66846 (PMC12046252; doi:10.2196/66846)
Supplement: Multimedia Appendix 1 [file resprot_v14i1e66846_app1.pdf]

The UNIVERSITY OF CHICAGO

The Division of the Biological Sciences • The University of Chicago Medical Center

VERBAL CONSENT TO PARTICIPATE IN A RESEARCH STUDY

IRB23-0385

A pilot to test Chronic Kidney Disease education program for hospitalized African American patients (I-C-CKD)

You are being contacted because you participated in the Hospitalist Project (HP) study at the University of Chicago, IRB 16-1131, and are African American adults with stage 3b or above CKD with an expected stay of greater than 48 hrs. The purpose of this study is to evaluate computerized educational materials for patients with chronic kidney disease. We plan to educate patients about renal replacement therapy options and to assist in developing and implementing a plan around renal replacement therapy. We want to study what type of education program can best help patients learn about kidney disease and make the decisions that are best for them.

This study is a randomized control trial. This means that you will be assigned to either the experimental group or control group, both have different educational course content. The experimental group is the group that receives the intervention, in this case it will include specific education materials, and the control group receives the standard care. The standard care will be the education materials the hospital already provides to educate you on your health. Both are in online format, and you will not know what group you are in during this study.

The odds of being enrolled in the experiment or control group are 50/50, this is similar to the flipping a coin.

If you decide to participate in the study, we will ask you to do the following things:

• Take a 10–20-minute online survey on a computer with one of our research assistants before you review some education materials. This computer survey will ask you questions regarding your knowledge of chronic kidney disease and your preferences for treatment if your kidneys were to fail.

• Review either the control or experimental patient education materials for about 15-20 minutes.

• Take another online post-education survey on the computer with the research assistant after you review the education materials.

• You will then be provided with additional questions about goal setting (5 minutes). These additional questions are the same for each group.

• Participate in one additional follow-up survey (5-10 minutes) at 1-month after your visit. Our research assistant will call you to ask some follow-up questions about your health-related visits and preferences for treatment.

• We will need to collect your name, address, and telephone number in order to ask you to complete the follow-up survey 1-month after your visit.

As a part of the Hospitalist Project, you have previously allowed for data to be collected from your medical records for the purposes of research which may be used in this study. We will use your

medical record and survey information already collection by the Hospitalist Project. We are interested in demographic information, health literacy and quality of life.

You will be in the study until the completion of the 1-month follow-up survey or up to 45 days after your initial survey. We will keep the data for 5 years after the study is completed.

We may decide to end this interview without your consent if:

• You are unable to meet the requirements of the study;

• Your medical condition changes;

• New information becomes available that indicates that participation in this study is not in your best interest.

In this study, you are being asked to answer some questions regarding your knowledge about your kidney disease and your preferences for treatment. You may feel uncomfortable answering some of the questions during the in-person surveys or follow-up calls. You do not have to answer any questions that make you feel uncomfortable. There is a risk of emotional distress as a result of discussing your personal experiences. We hope you will learn more about your health and different treatments for CKD. We hope that the program we are developing will help improve the quality of life for other African American patients with CKD.

You will receive a $25 gift card at the end of in-person surveys and a $25 gift card for the follow-up call at 30 days ($50 value total). If a scheduling conflict occurs and you are unable to complete the in-person surveys, you will still receive a $10 gift card for your time. You will receive the gift card in person for the study visit and we will mail the gift card to you after completing the follow-up survey.

If you have further questions about the study, you may call the principal investigator, Dr. Milda Saunders (773) 702-5941.

If you have any questions concerning your rights in this research study you may contact the Institutional Review Board, which is concerned with the protection of subjects in research projects. You may reach the Committee office between 8:30 am and 5:00 pm, Monday through Friday, by calling (773) 702-6505 or by writing:

Institutional Review Board, University of Chicago, 5841 S. Maryland Ave., I-625, MC7132, Chicago, IL 60637.

Your participation in this research is voluntary, and you will not be penalized or lose benefits if you refuse to participate or decide to stop. You may still receive the educational materials from the control group if you decide not to participate in this study, as this is the standard of care. Do you consent to participate in this project?
